# Supplementary material for: Brain leptin reduces liver lipids by increasing hepatic triglyceride secretion and lowering lipogenesis
Source: Nat Commun. 2019 Jun 20;10:2717. doi: 10.1038/s41467-019-10684-1 (PMC6586634; doi:10.1038/s41467-019-10684-1)
Supplement: Supplementary file 1 — Supplementary Information [file 41467_2019_10684_MOESM1_ESM.pdf]

Supplementary information

Brain leptin reduces liver lipids by increasing hepatic triglyceride secretion and lowering lipogenesis

Hackl et. al

Supplementary Figures

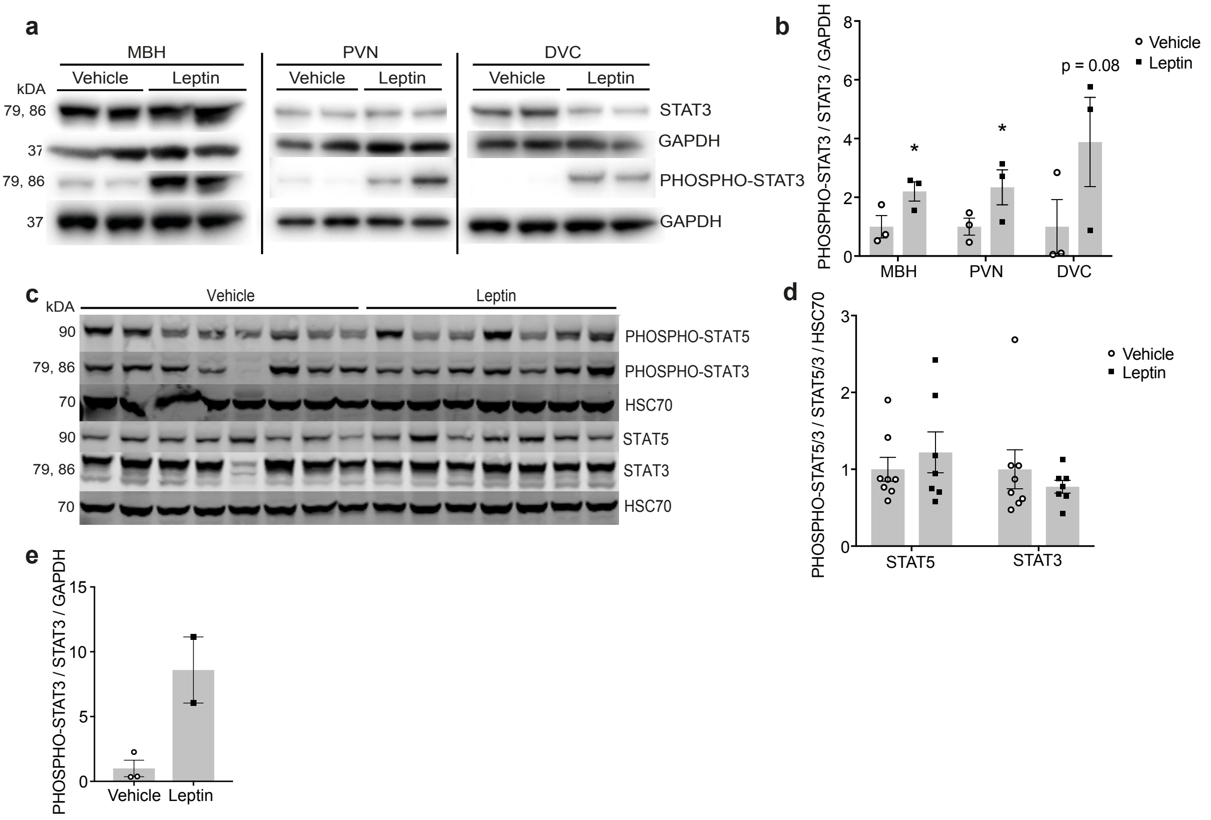

Supplementary Figure 1 | A leptin injection into the 3rd ventricle activates STAT3 signaling in the hypothalamus and the brainstem, but not in the liver.

(a) Representative Western blot analyses of STAT3, phospho-STAT3 (Tyr705) and GAPDH of brain punch biopsies of the mediobasal hypothalamus (MBH), the paraventricular nucleus and the dorsal vagal complex (DVC) from rats that were injected with either leptin (10 µg in 10 µl) or vehicle into the 3rd ventricle (ICV). Note that, lanes were run on the same gel but were non-contiguous. (b) Quantification of Western blot analyses in (a) of phospho-STAT3 (Tyr705) normalized to total STAT3 (n = 3 per group). (c) Western blots of STAT5, phospho-STAT5 (Tyr694), STAT3, phospho-STAT3 (Tyr705) and HSC70 of liver samples of acute ICV leptin infused animals compared to vehicle infused controls. (d) Quantification of Western blot analyses corresponding to (c) normalized to the housekeeping protein HSC70 and the respective STAT total protein levels (n ≥ 7 per group). (e) Quantification of Western blot analyses of phospho-STAT3 (Tyr705) normalized to total STAT3 of punch biopsies of the DVC after acute intranasal leptin/vehicle instillation in fasted male rats. (n ≥ 2 per group). All data are mean ± SEM; * $p < 0.05$ vs vehicle group by one-tailed Student’s t-test in (a) and (b) and two-tailed Student’s t-test in (c) and (d); open circles: ICV vehicle; black squares: ICV leptin.

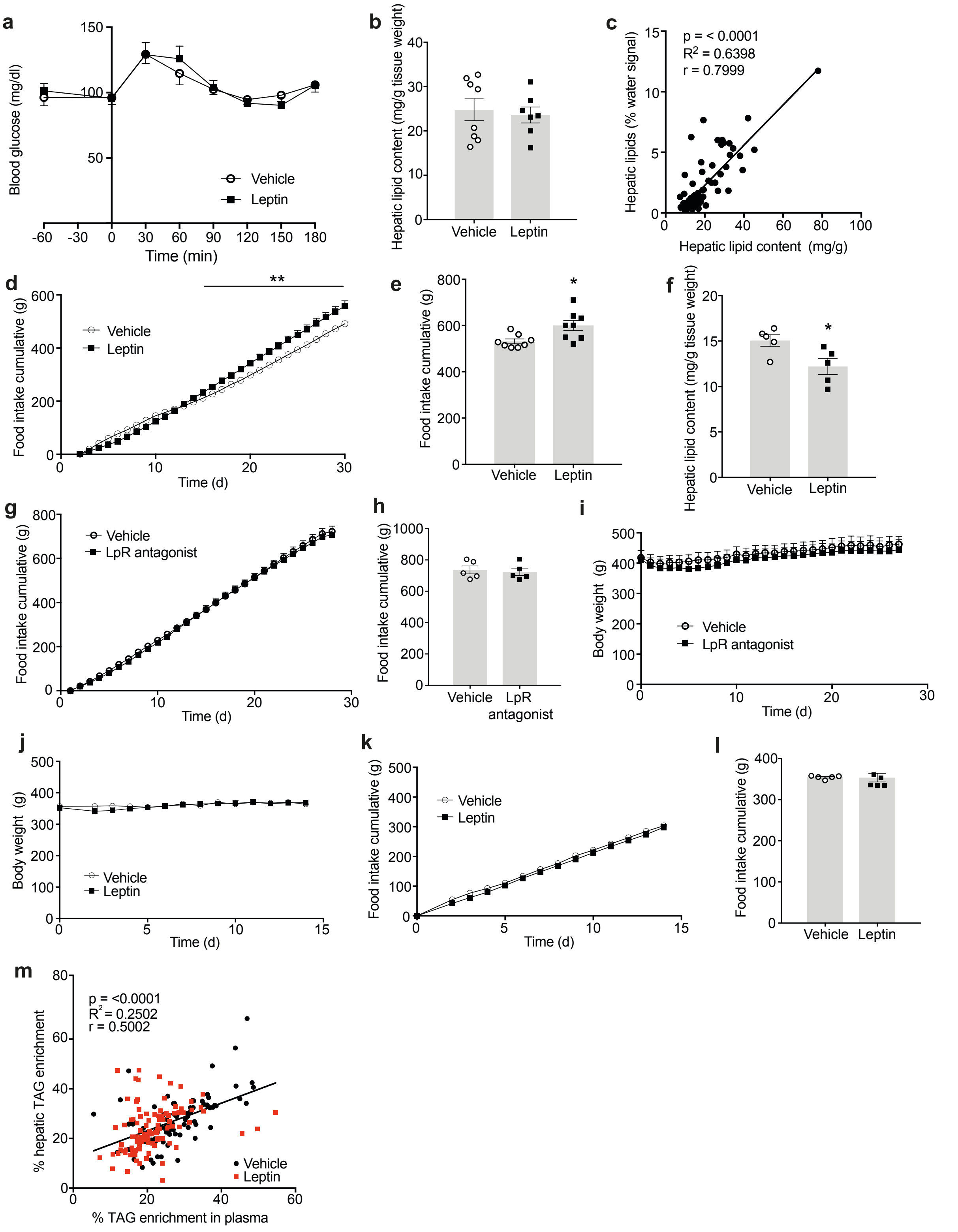

Supplementary Figure 2 | Blood glucose levels in the acute tyloxapol experiments are not different between groups. ^{1}H-MRS is an accurate method to non-invasively measure hepatic lipid content in rats. Food intake and body weight were not affected by a chronic low-dose leptin receptor (LpR) antagonist infusion.

(a) Blood glucose levels during the acute tyloxapol infusion studies ($n \geq 7$; see protocol in Figure 1a). (b) Hepatic lipid content assessed after a Folch extraction following an acute 4 hr leptin/vehicle infusion. (c) Liver fat data assessed by ^{1}H–MRS from our chronic intracerebroventricular (ICV) infusion experiments correlate with conventional liver triglyceride measurements using chloroform-methanol (Folch) extraction (total data points = 78). This verifies the accuracy of the ^{1}H-magnetic resonance spectroscopy (^{1}H-MRS) method in rodents. (d) Cumulative food intake over 28 days in rats during a chronic ICV leptin/vehicle infusion (see protocol in Figure 1f). Note that the vehicle group was food restricted to match the body weight of the ICV leptin group (n = 8 per goup). (e) Cumulative food intake after 28 days (final timepoint in Supplementary Figure 2d; n = 8 per group). (f) Hepatic lipid content assessed with Folch extraction after 28 days of an ICV leptin/vehicle infusion. Note that the ^{1}H-MRS data from Figure 1h match the results from the conventional Folch extraction (n = 5 per group). (g) Cumulative food intake over 28 days during a leptin receptor antagonist (LpR antagonist) or vehicle infusion (n = 5) (see protocol in Figure 1l). (h) Cumulative food intake after 28 days (final timepoint in Supplementary Figure 2g; n = 5 per group) and (i) body weight curves during the chronic LpR antagonist infusion study (n = 5 per group). (j) Body weights and (k) cumulative food intake over 14 days in rats during a chronic ICV leptin/vehicle infusion accompanied by 6 % D_2O in the drinking water (see protocol in Figure 3i). (l) Cumulative food intake after 14 days (final timepoint in Supplementary Figure 2k; $n \geq 5$ per group). (m) Correlation of liver TAG enrichment vs. plasma TAG enrichment across the detected lipid species and treatment groups ($n \geq 4$ animals per group, total data points = 192). All data are mean ± SEM; * $p < 0.05$, ** $p < 0.01$ vs vehicle group by two-tailed Student's t-test; (a-f and j-l): open circles: ICV vehicle; black squares: ICV leptin; (g-i): open circles: ICV vehicle; black squares: ICV leptin receptor antagonist; (m): black circles: ICV vehicle; red squares: ICV leptin.

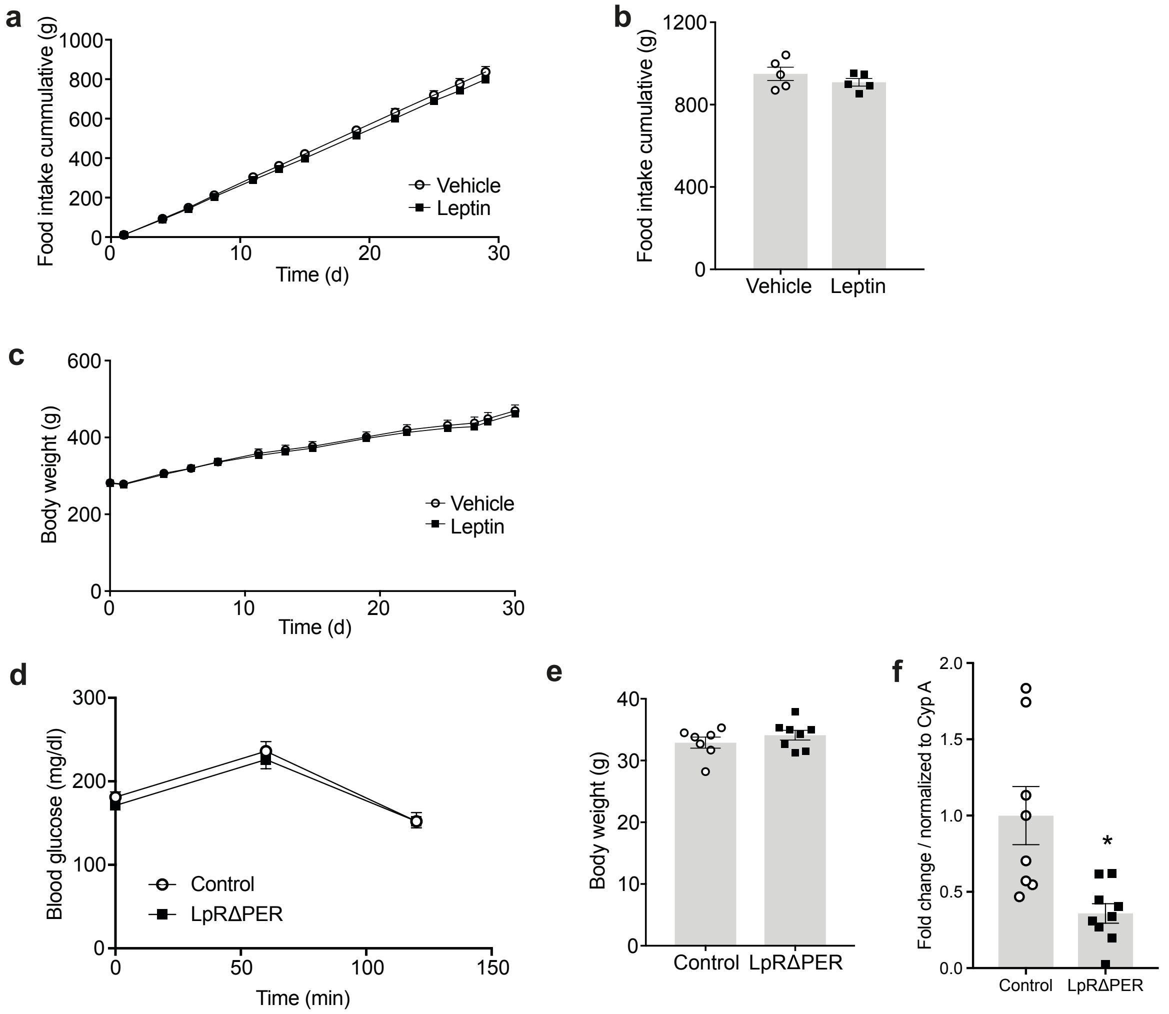

Supplementary Figure 3 | LpRΔPER mice have similar body weights compared to control mice. A chronic IP (intraperitoneal) infusion of leptin at a dose that shows metabolic effects when infused centrally (ICV) does neither affect body weight nor food intake.

(a) Cumulative food intake over 28 days in rats that received leptin or vehicle systemically via a chronic IP infusion (n = 5 per group; see protocol in Figure 2a). The IP leptin dose matched that of the ICV leptin infusion experiments. (b) Cumulative food intake after 28 days (final timepoint in Supplementary Figure 3a). (c) Body weights during the IP leptin/vehicle infusion experiment (n = 5 per group). (d) Blood glucose levels of tamoxifen-induced LpRΔPER and control mice during an acute tyloxapol experiment (n ≥ 7 per group). (e) Mean body weight of LpRΔPER and control mice (n ≥ 7 per group). (f) LpR mRNA expression after tamoxifen induction in the livers of LpRΔPER and control mice as assessed by SYBR Green PCR (n ≥ 7

per group); All data are mean ± SEM * $p < 0.05$ vs vehicle group by two-tailed Student's t-test; (a-c): open circles: IP vehicle; black squares: IP leptin; (d-f): open circles: controls; black squares: tamoxifen-inducible leptin receptor knock out (LpRΔPER) mice.

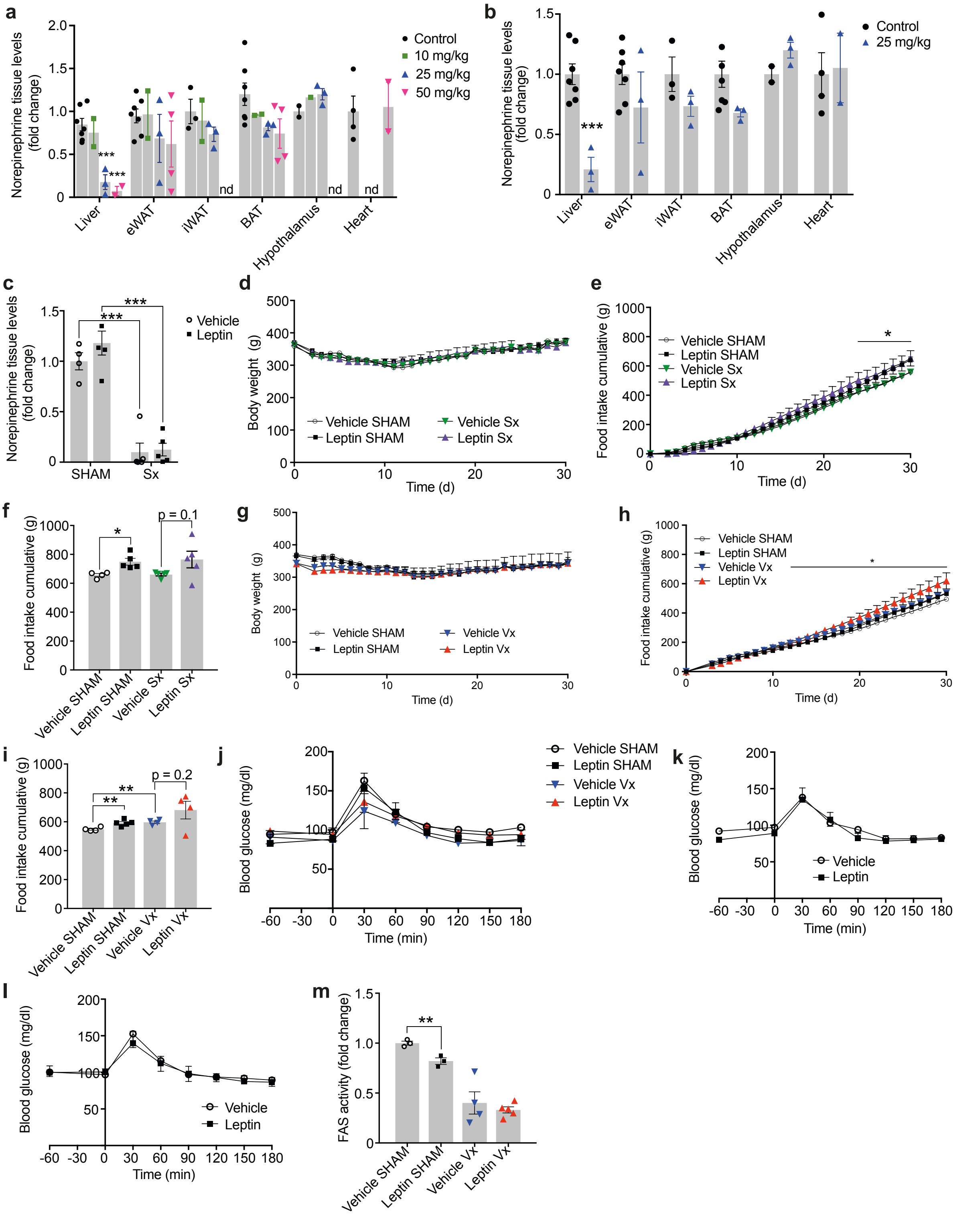

Supplementary Figure 4 | An intraportal 6-hydroxydopamine (6-OHDA) bolus injection reduces hepatic, but not extrahepatic norepinephrine levels. Brain leptin fails to reduce FAS activity in vagotomized livers.

(a) Dose finding study – tissue norepinephrine levels after an intraportal bolus injection of different doses of the sympathotoxin 6-OHDA or vehicle. Due to a significant first pass effect

an intraportal 6-OHDA dose between 25 and 50 mg per kg body weight induces > 80 % reduction in hepatic norepinephrine (n ≥ 3 per group), whereas white and brown adipose tissue, heart and brain are not significantly affected. Animals were analyzed 8 days after the injection. (b) Dose verification study – norepinephrine levels in various tissues after a bolus injection of 25 mg per kg body weight 6-OHDA or vehicle into the portal vein (n ≥ 3 per group). Animals were analyzed 32 days after the injection. (c) Liver norepinephrine levels after 28 days of intracerebroventricular (ICV) leptin/vehicle infusion in rats subject to intraportal 25 mg/kg body weight 6-OHDA injection or vehicle (n ≥ 4 per group). (d) Body weight during 28 days of chronic ICV leptin/vehicle infusion in rats after a liver specific sympathectomy (Sx) or a sham procedure (n ≥ 4 per group, see protocol in Figure 4a). (e) Cumulative food intake over 28 days during the ICV leptin/vehicle infusion experiment in liver sympathectomized rats or sham controls (n ≥ 4 per group). Note that the rats were weight matched by calorically restricted feeding of the controls. (f) Cumulative food intake after 28 days (final timepoint in Supplementary Figure 4e; n ≥ 4 per group). (g) Body weight curves during 28 days of a chronic ICV leptin/vehicle infusion study in rats after cutting the hepatic branch of the vagus nerve (liver vagotomy, Vx) and sham manipulated controls (n ≥ 4 per group, see protocol in Figure 4a). (h) Cumulative food intake over 28 days in rats subjected to ICV leptin/vehicle infusion after liver vagotomy or sham procedure. Note that the rats were weight matched by restricted feeding of the controls. (i) Cumulative food intake after 28 days (final timepoint in Supplementary Figure 4h; n ≥ 4 per group). (j-l) Blood glucose levels during acute tyloxapol infusion studies. Depicted are results from (j) animals after liver vagotomy or a sham procedure that received either ICV leptin or vehicle (n ≥ 3 per group), (k) rats that received a leptin or vehicle injection directly into the mediobasal hypothalamus (MBH; n ≥ 5 per group), or (l) rats that received a leptin or vehicle injection directly into the dorsal vagal complex (DVC; n ≥ 4 per group). (m) Liver FAS activity after an acute ICV leptin/vehicle infusion in rats after

surgical liver vagotomy or sham procedure (see protocol in Figure 4d; $n \geq 3$ per group). All data are mean ± SEM; * $p < 0.05$; ** $p < 0.01$; *** $p < 0.001$ vs vehicle or vehicle sham group by two-tailed Student's t-test; n.d. not done; (a-b): black circles: control; green squares, blue and pink triangles: respective 6-OHDA dose used; (c-m): open circles: ICV vehicle; black squares: ICV leptin; green triangles pointing down: ICV vehicle plus liver Sx; violet triangles pointing up: ICV leptin plus liver Sx; blue triangles pointing down: ICV vehicle plus liver Vx; red triangles pointing up: ICV leptin plus liver Vx.

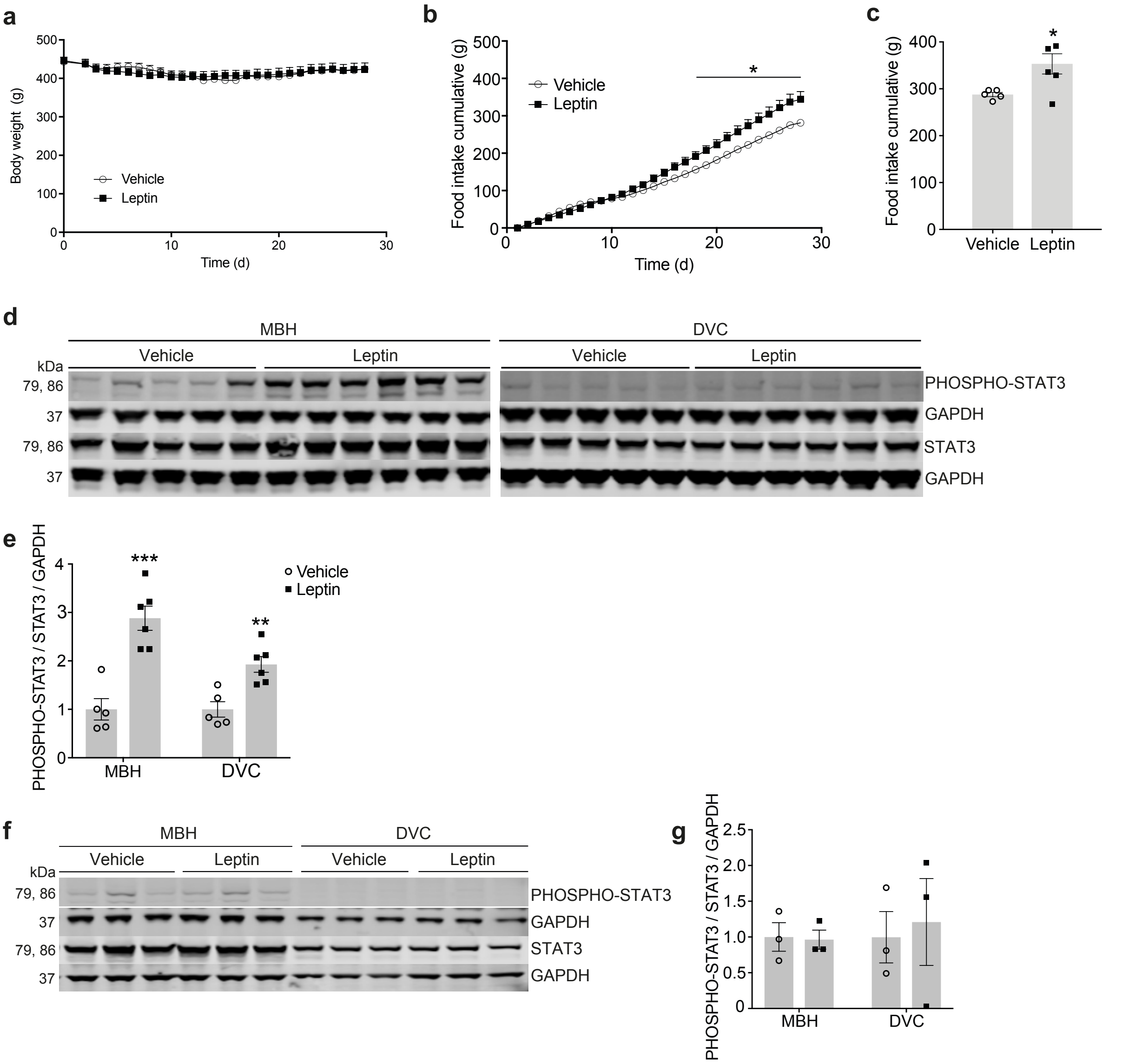

Supplementary Figure 5 | The chronic ICV leptin infused rats on a HFD had similar body weights compared to vehicle infused controls. Chronic ICV leptin induces brain STAT3 signaling even in the obese state.

(a) Body weight curves over 28 days during a 4-week chronic leptin/vehicle infusion in rats that were fed a 60 % high-fat diet 4 weeks before, and for the 4 weeks during the ICV leptin/vehicle infusion study (see protocol in Figure 5a). Note that due to the anticipated brain leptin resistance in these rats and the approximately 3 to 4 times higher peripheral leptin levels after 8 weeks of HFD feeding compared to regular chow (see leptin levels in Supplementary Table 1 vs. Supplementary Table 5) the ICV leptin dose was tripled. (b) Cumulative food intake over the course of the 28 days. Notably, the vehicle group was food restricted to match the body weight of the ICV leptin group. (c) Cumulative food intake after 28 days (final timepoint in Supplementary Figure 5b; n ≥ 5 per group); (d) Western blot analyses of STAT3, phospho-

STAT3 (Tyr705) and GAPDH of biopsies of the mediobasal hypothalamus (MBH) and the dorsal vagal complex (DVC) of animals on a HFD diet subjected to a chronic ICV leptin/vehicle infusion (see protocol in Figure 5a) (e) Quantification of Western blot analyses of phospho-STAT3 in (d) normalized to total STAT3 ($n \geq 5$ per group). (f) Western blot analyses of STAT3, phospho-STAT3 (Tyr705) and GAPDH of punch biopsies of the MBH and the DVC 1 hour after an IP leptin (1 mg/kg body weight) or saline injection in 8-week HFD fed rats. (g) Quantification of Western blot analyses of phospho-STAT3 in (f) normalized to total STAT3 ($n = 3$ per group). All data are mean ± SEM; * $p < 0.05$; ** $p < 0.01$; ***$p < 0.001$ vs vehicle group by two-tailed Student's t-test; open circles: ICV vehicle; black squares: ICV leptin.

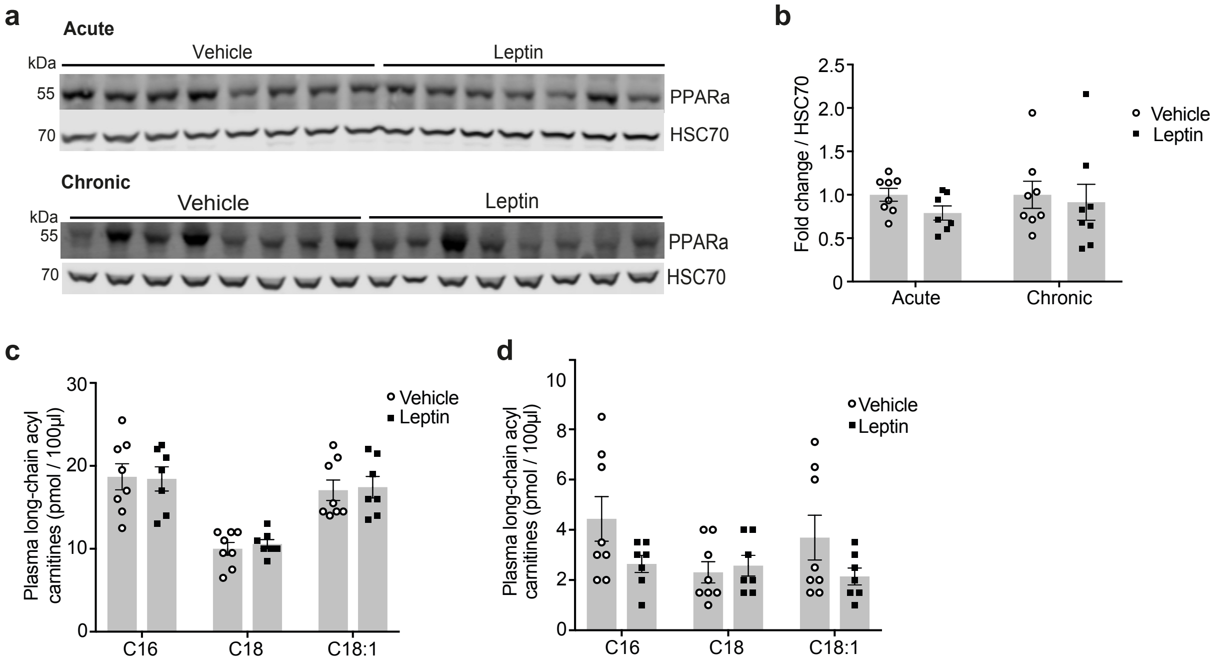

Supplementary Figure 6 | Brain leptin does not affect PPARα expression and circulating long-chain acyl-carnitines.

(a) Western blot analyses of PPARα protein expression in liver tissue of acute and chronic ICV leptin/vehicle infused animals (b) Quantification of Western blot analysis of PPARα in (a) (n ≥ 7 per group). (c, d) Plasma lipid-derived long-chain acyl-carnitines of acute (c) and chronic (d) ICV leptin/vehicle infused rats (n ≥ 7 per group). All data are mean ± SEM; open circles: ICV vehicle; black squares: ICV leptin.

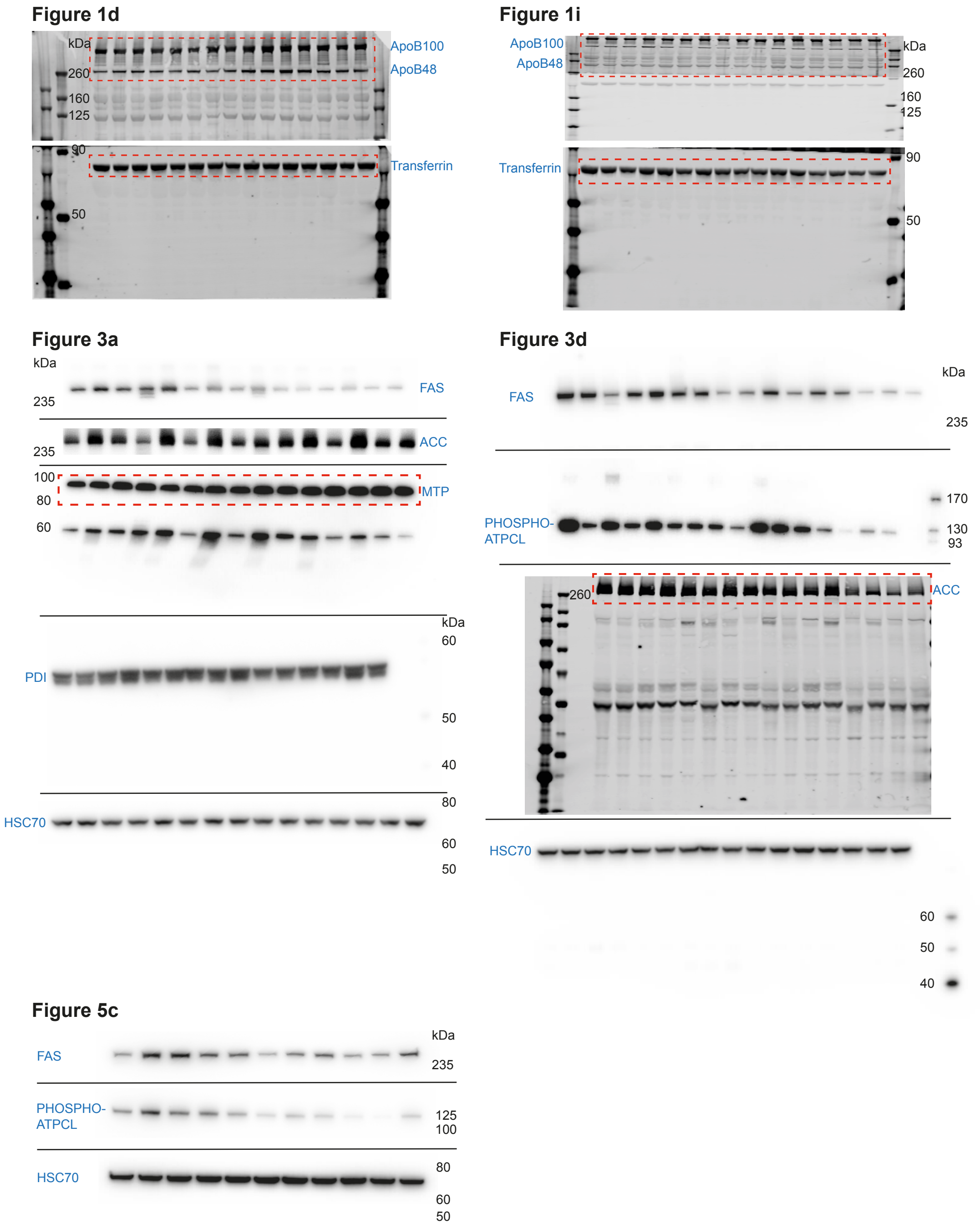

Supplementary Figure 7 | Fully uncropped scans of all blots presented in the main figures.

Lanes used in figures are marked by dashed boxes when more than one lane is present and the antibodies used for detection are indicated. To allow concomitant detection of more than one protein on the same membrane, membranes were cut with a scalpel.

Supplementary Tables

Supplementary Table 1: Supporting blood parameters and characteristics of ICV infusion experiments. All data are mean ± SEM.

		Treatment				p vs. Vehicle	n
ICV leptin/vehicle *acute*	Plasma insulin (mU/l)	Vehicle	4.70	±	1.90		8
		Leptin	3.30	±	1.10	0.518	7
	Free fatty acids (µM)	Vehicle	692	±	116		8
		Leptin	542	±	85	0.310	7
	Plasma leptin (pg/ml)	Vehicle	180	±	21		8
		Leptin	225	±	22	0.140	7
	Body weight (g)	Vehicle	332	±	14.1		13
		Leptin	341	±	13.8	0.531	12
	Plasma free glycerol (mg/ml)	Vehicle	1.72	±	0.32		8
		Leptin	1.70	±	0.14	0.952	7
	Plasma ketone bodies (µmol/l)	Vehicle	551	±	64		8
		Leptin	570	±	43	0.810	7
ICV leptin/vehicle *chronic*	Plasma glucose (mg/dl)	Vehicle	157.7	±	12.4		8
		Leptin	98.6	±	18.8	0.020	8
	Plasma insulin (mU/l)	Vehicle	9.90	±	1.50		8
		Leptin	1.80	±	0.80	0.0003	8
	Free fatty acids (µM)	Vehicle	271	±	28		8
		Leptin	95	±	17	0.0001	8
	Plasma TGs (mg/ml)	Vehicle	0.42	±	0.06		8
		Leptin	0.15	±	0.05	0.005	8
	Plasma leptin (pg/ml)	Vehicle	157	±	16		8
		Leptin	92	±	14	0.010	8
	Plasma ketone bodies (µmol/l)	Vehicle	224	±	37		8
		Leptin	121	±	9	0.018	8
	Initial hepatic lipids (% water signal)	Vehicle	0.84	±	0.04		8
		Leptin	0.84	±	0.06	0.960	8
	eWAT fat pad weight (g)	Vehicle	2.01	±	0.21		8
		Leptin	0.45	±	0.23	0.0002	8
ICV LpR antagonist	Blood glucose (mg/dl)	Vehicle	82.3	±	3.6		5
		LpR inhibitor	83.1	±	1.7	0.807	5
	Plasma insulin (mU/l)	Vehicle	8.60	±	1.20		5
		LpR inhibitor	8.80	±	1.80	0.943	5
	Free fatty acids (µM)	Vehicle	300	±	14		5
		LpR inhibitor	311	±	19	0.765	5
	Plasma TGs (mg/ml)	Vehicle	0.51	±	0.06		5
		LpR inhibitor	0.61	±	0.07	0.294	5
	Plasma leptin (pg/ml)	Vehicle	203	±	23		5
		LpR inhibitor	210	±	28	0.843	5
ICV leptin/vehicle + 6 % D_2O	Blood glucose (mg/dl)	Vehicle	80.2	±	2.29		5
		Leptin	74.1	±	7.49	0.438	6
	Free fatty acids (µM)	Vehicle	303	±	20		5
		Leptin	297	±	20	0.925	6
	Plasma ketone bodies (µmol/l)	Vehicle	398	±	34		5
		Leptin	237	±	32	0.007	6

Supplementary Table 2: Supporting blood parameters and characteristics of IP leptin/vehicle infused animals and LpRΔPER knock-out and control mice. All data are mean ± SEM.

		Treatment				p vs. Vehicle	n
Leptin/vehicle IP *chronic*	Blood glucose (mg/dl)	Vehicle	90.3	±	3.3		5
		Leptin	92.7	±	3.0	0.601	5
	Plasma insulin (mU/l)	Vehicle	9.50	±	0.80		5
		Leptin	9.00	±	1.70	0.621	5
	Free fatty acids (µM)	Vehicle	325	±	19		5
		Leptin	360	±	27	0.316	5
	Plasma TGs (mg/ml)	Vehicle	0.66	±	0.07		5
		Leptin	0.76	±	0.11	0.504	5
	Plasma leptin (pg/ml)	Vehicle	281	±	19		5
		Leptin	386	±	89	0.282	5
LpRΔPER KO mice	Age (weeks)	Controls	24.9	±	2.3		7
		LpR KO mice	22.5	±	1.3	0.326	8
	Body weight (g)	Controls	32.9	±	0.9		7
		LpR KO mice	34.1	±	0.8	0.377	8

Supplementary Table 3: Supporting blood parameters and characteristics of denervation experiments. All data are mean ± SEM.

		Treatment		p vs. Vehicle	n
Sx/sham + ICV leptin/vehicle *chronic*	Blood glucose (mg/dl)	Vehicle SHAM	89.0 ± 0.9		4
		Leptin SHAM	75.1 ± 2.8	0.004	5
		Vehicle Sx	82.3 ± 4.0		5
		Leptin Sx	76.9 ± 5.4	0.442	5
	Plasma insulin (mU/l)	Vehicle SHAM	5.19 ± 1.13		3
		Leptin SHAM	1.60 ± 0.36	0.010	5
		Vehicle Sx	3.23 ± 0.82		5
		Leptin Sx	2.47 ± 1.30	0.638	5
	Free fatty acids (uM)	Vehicle SHAM	327 ± 66		4
		Leptin SHAM	235 ± 17	0.178	5
		Vehicle Sx	280 ± 19		5
		Leptin Sx	236 ± 52	0.451	5
	Plasma TGs (mg/ml)	Vehicle SHAM	0.36 ± 0.01		4
		Leptin SHAM	0.25 ± 0.02	0.002	5
		Vehicle Sx	0.46 ± 0.12		5
		Leptin Sx	0.20 ± 0.05	0.076	5
	Plasma leptin (pg/ml)	Vehicle SHAM	209 ± 9		4
		Leptin SHAM	162 ± 6	0.003	5
		Vehicle Sx	211 ± 16		5
		Leptin Sx	190 ± 30	0.040	4
Vx/sham + ICV leptin/vehicle *chronic*	Blood glucose (mg/dl)	Vehicle SHAM	83.6 ± 2.9		4
		Leptin SHAM	77.7 ± 1.8	0.112	5
		Vehicle Vx	82.3 ± 2.6		4
		Leptin Vx	72.9 ± 11.0	0.439	4
	Plasma insulin (mU/l)	Vehicle SHAM	8.51 ± 0.71		4
		Leptin SHAM	4.83 ± 1.75	0.120	5
		Vehicle Vx	9.20 ± 1.99		4
		Leptin Vx	4.99 ± 1.87	0.128	4
	Free fatty acids (uM)	Vehicle SHAM	350 ± 79		4
		Leptin SHAM	341 ± 33	0.912	5
		Vehicle Vx	305 ± 9		4
		Leptin Vx	268 ± 26	0.2101	4
	Plasma TGs (mg/ml)	Vehicle SHAM	0.36 ± 0.01		4
		Leptin SHAM	0.26 ± 0.04	0.074	5
		Vehicle Vx	0.41 ± 0.05		4
		Leptin Vx	0.23 ± 0.03	0.018	4
	Plasma leptin (pg/ml)	Vehicle SHAM	220 ± 17		4
		Leptin SHAM	211 ± 18	0.740	5
		Vehicle Vx	275 ± 64		4
		Leptin Vx	197 ± 23	0.292	4

Supplementary Table 4: Supporting blood parameters and characteristics of hepatic branch vagotomized animals following acute ICV leptin/vehicle infusion and acute DVC or MBH leptin/vehicle infused animals. All data are mean ± SEM.

		Treatment		p vs. Vehicle	n
Vx/sham + ICV leptin/vehicle *acute*	Plasma insulin (mU/l)	Vehicle SHAM	0.49 ± 0.03		3
		Leptin SHAM	3.43 ± 2.03	0.222	3
		Vehicle Vx	4.19 ± 1.78		4
		Leptin Vx	6.83 ± 2.83	0.484	5
	Free fatty acids (uM)	Vehicle SHAM	603 ± 24		3
		Leptin SHAM	538 ± 52	0.318	3
		Vehicle Vx	506 ± 22		4
		Leptin Vx	488 ± 63	0.817	5
	Plasma leptin (pg/ml)	Vehicle SHAM	138 ± 2		3
		Leptin SHAM	166 ± 14	0.119	3
		Vehicle Vx	138 ± 1		4
		Leptin Vx	171 ± 35	0.435	5
	Body weight (g)	Vehicle SHAM	418 ± 15.5		3
		Leptin SHAM	472 ± 18.0	0.086	3
		Vehicle Vx	416 ± 13.1		4
		Leptin Vx	389 ± 3.5	0.065	5
DVC leptin/vehicle *acute*	Plasma insulin (mU/l)	Vehicle	1.83 ± 0.57		7
		Leptin	3.15 ± 1.71	0.421	5
	Free fatty acids (uM)	Vehicle	554 ± 31		7
		Leptin	534 ± 35	0.690	5
	Plasma leptin (pg/ml)	Vehicle	142 ± 2		7
		Leptin	142 ± 2	0.936	5
	Body weight (g)	Vehicle	393 ± 4.8		7
		Leptin	404 ± 13.9	0.396	5
MBH leptin/vehicle *acute*	Plasma insulin (mU/l)	Vehicle	3.71 ± 0.87		3
		Leptin	3.40 ± 0.82	0.808	4
	Free fatty acids (uM)	Vehicle	668 ± 61		3
		Leptin	582 ± 25	0.200	4
	Plasma leptin (pg/ml)	Vehicle	137 ± 2		3
		Leptin	143 ± 6	0.440	4
	Body weight (g)	Vehicle	408 ± 7.6		3
		Leptin	396 ± 8.3	0.360	4

Supplementary Table 5: Supporting blood parameters and characteristics HFD fed animals. All data are mean ± SEM.

		Treatment				p vs. Vehicle	n
High-fat diet + ICV leptin/vehicle *chronic*	Plasma glucose (mg/dl)	Vehicle	146.4	±	20.8		5
		Leptin	110.9	±	19.1	0.241	6
	Plasma insulin (mU/l)	Vehicle	8.60	±	1.40		5
		Leptin	4.20	±	2.20	0.141	6
	Free fatty acids (uM)	Vehicle	191	±	43		5
		Leptin	155	±	29	0.488	6
	Plasma TGs (mg/ml)	Vehicle	0.37	±	0.10		5
		Leptin	0.14	±	0.04	0.055	6
	Plasma leptin (pg/ml)	Vehicle	557	±	137		5
		Leptin	159	±	23	0.012	6
	eWAT fat pad weight (g)	Vehicle	4.26	±	0.41		5
		Leptin	2.41	±	0.73	0.068	6

.
