# Supplementary material for: Brain leptin reduces liver lipids by increasing hepatic triglyceride secretion and lowering lipogenesis
Source: Nat Commun. 2019 Jun 20;10:2717. doi: 10.1038/s41467-019-10684-1 (PMC6586634; doi:10.1038/s41467-019-10684-1)
Supplement: Supplementary file 3 — Reporting Summary [file 41467_2019_10684_MOESM3_ESM.pdf]

nature research

Corresponding author(s): Thomas Scherer

Last updated by author(s): Jan 17, 2019

Reporting Summary

Nature Research wishes to improve the reproducibility of the work that we publish. This form provides structure for consistency and transparency in reporting. For further information on Nature Research policies, see Authors & Referees and the Editorial Policy Checklist.

Statistics

For all statistical analyses, confirm that the following items are present in the figure legend, table legend, main text, or Methods section.

n/a	Confirmed	
☐	☒	The exact sample size (*n*) for each experimental group/condition, given as a discrete number and unit of measurement
☒	☐	A statement on whether measurements were taken from distinct samples or whether the same sample was measured repeatedly
☐	☒	The statistical test(s) used AND whether they are one- or two-sided<br>*Only common tests should be described solely by name; describe more complex techniques in the Methods section.*
☒	☐	A description of all covariates tested
☒	☐	A description of any assumptions or corrections, such as tests of normality and adjustment for multiple comparisons
☐	☒	A full description of the statistical parameters including central tendency (e.g. means) or other basic estimates (e.g. regression coefficient) AND variation (e.g. standard deviation) or associated estimates of uncertainty (e.g. confidence intervals)
☐	☒	For null hypothesis testing, the test statistic (e.g. *F*, *t*, *r*) with confidence intervals, effect sizes, degrees of freedom and *P* value noted<br>*Give P values as exact values whenever suitable.*
☒	☐	For Bayesian analysis, information on the choice of priors and Markov chain Monte Carlo settings
☒	☐	For hierarchical and complex designs, identification of the appropriate level for tests and full reporting of outcomes
☐	☒	Estimates of effect sizes (e.g. Cohen's *d*, Pearson's *r*), indicating how they were calculated

Our web collection on statistics for biologists contains articles on many of the points above.

Software and code

Policy information about availability of computer code

Data collection	Quantification of western blots was performed with Image J (Version 1.48v) and LI-COR ImageStudioLite (Version 5.2.5)
Data analysis	GraphPad Prism (Version 7) and MS Excel

For manuscripts utilizing custom algorithms or software that are central to the research but not yet described in published literature, software must be made available to editors/reviewers. We strongly encourage code deposition in a community repository (e.g. GitHub). See the Nature Research guidelines for submitting code & software for further information.

Data

Policy information about availability of data

All manuscripts must include a data availability statement. This statement should provide the following information, where applicable:

- Accession codes, unique identifiers, or web links for publicly available datasets
- A list of figures that have associated raw data
- A description of any restrictions on data availability

A Reporting Summary for this Article is available as a Supplementary Information file. Uncropped scans of all western blots presented in the main figures are provided in the Supplementary Information. All data supporting the findings of this study are available from the corresponding author on request.

Field-specific reporting

Please select the one below that is the best fit for your research. If you are not sure, read the appropriate sections before making your selection.

☒ Life sciences ☐ Behavioural & social sciences ☐ Ecological, evolutionary & environmental sciences

Life sciences study design

All studies must disclose on these points even when the disclosure is negative.

Sample size	Sample size was estimated on the basis of similar studies performed by our group and reports from the literature. Conventional sample sizes in exploratory basic metabolic research were used.
Data exclusions	In the chronic infusion experiment with the leptin receptor antagonist (Fig. 1m) we excluded one rat in the leptin receptor antagonist group due to impaired recovery after surgery due to overgrown teeth and malocclusion.
Replication	All experiments were at least repeated three times. All replications were successful and supported the final conclusions.
Randomization	There was no randomization of animals.
Blinding	Investigators were not blinded.

Reporting for specific materials, systems and methods

We require information from authors about some types of materials, experimental systems and methods used in many studies. Here, indicate whether each material, system or method listed is relevant to your study. If you are not sure if a list item applies to your research, read the appropriate section before selecting a response.

Materials & experimental systems

n/a	Involved in the study	
☐	☒	Antibodies
☒	☐	Eukaryotic cell lines
☒	☐	Palaeontology
☐	☒	Animals and other organisms
☒	☐	Human research participants
☒	☐	Clinical data

Methods

n/a	Involved in the study	
☒	☐	ChIP-seq
☒	☐	Flow cytometry
☒	☐	MRI-based neuroimaging

Antibodies

Antibodies used	Primary antibodies against ACC (Cat. 3676, Lot #6) phospho-ATPCL Ser455 (Cat. 4331, Lot #2), PDI (Cat. 3501, Lot #3), GAPDH (Cat. 5174, Lot #4), STAT3 total (Cat. 4904, Lot #7) and phospho-Stat3 Tyr705 (Cat. 9145, Lot #26) (all Cell Signaling Technology); FAS (Cat. 610962, Lot #3266988), MTP (Cat. 612022, Lot #3266983) (both BD Bioscience); PPARα (Cat. sc-398394, Lot #G0571), STAT5 total (Cat. sc-74442, Lot #C1618) and HSC70 (Cat. sc-7298, Lot #E1914) (all Santa Cruz Biotechnology); phospho-STAT5 alpha Tyr694 (Cat. 71-6900, Lot #TF268386) (Thermo Fisher Scientific); Transferrin (Cat. ab82411; Abcam) and ApoB anti-serum (a gift from Dr. Larry Swift; Vanderbilt University Medical Center) were used.
Validation	All antibodies were validated by the manufacturer.<br>Cell Signaling: https://www.cellsignal.com/contents/our-approach/cst-antibody-validation-principles/ourapproach-validation-principles; details for each specific antibody are available on https://www.cellsignal.com/<br>BD Bioscience: FAS: http://www.bdbiosciences.com/ds/pm/tds/610962.pdf; MTP: http://www.bdbiosciences.com/ds/pm/tds/612022.pdf<br>Santa Cruz Biotechnology: PPARα: https://datasheets.scbt.com/sc-398394.pdf; STAT5: https://datasheets.scbt.com/sc-74442.pdf; HSC70: https://datasheets.scbt.com/sc-7298.pdf<br>Abcam: https://www.abcam.com/primary-antibodies/improving-reproducibility-with-better-antibodies; Transferrin: https://www.abcam.com/transferrin-antibody-ab82411.html<br>Thermo Fisher: https://www.thermofisher.com/at/en/home/life-science/antibodies/invitrogen-antibody-validation.html?icid=ab-search-learning-ab-validation; phospho-STAT5: https://www.thermofisher.com/antibody/product/Phospho-STAT5-alpha-Tyr694-Antibody-Polyclonal/71-6900<br>ApoB anti-serum: validated in http://www.jbc.org/content/271/49/31491.long

Animals and other organisms

Policy information about studies involving animals; ARRIVE guidelines recommended for reporting animal research

Laboratory animals	10-week old male Sprague Dawley rats and LpRdeltaPER (LpRflox/flox Rosa26Cre-ERT2/+) and control mice (LpRflox/flox) as published in doi:en.2007-0261 [pii] 10.1210/en.2007-0261 were used in this study.
Wild animals	The study did not involve wild animals.

Field-collected samples	The study did not involve field-collected samples.
Ethics oversight	All experimental procedures were approved by the Austrian Federal Ministry of Science, Research, and Economy (BMWFW-66.009/0246-WF/V/3b/2015). Mouse experiments were approved by the International Animal Care and Use Committee of Mount Sinai School of Medicine, New York, NY (IACUC LA09-00174).

Note that full information on the approval of the study protocol must also be provided in the manuscript.
